# Supplementary material for: Dynamic transcriptomic profiles of zebrafish gills in response to zinc supplementation
Source: BMC Genomics. 2010 Oct 11;11:553. doi: 10.1186/1471-2164-11-553 (PMC3091702; doi:10.1186/1471-2164-11-553)
Supplement: Additional file 2 — Interactive Direct Interaction Network representing the molecular interactions between zinc, copper, iron, calcium and proteins encoded by transcripts changed by zinc supplementation. Mini web-site containing index.html and hyperlinked pages in subdirectory describing a Direct Interaction Network automatically generated based on curated interactions contained within the proprietary PathwayArchitect database. Ovals represent proteins and the circles symbolize metal ions. Objects are coloured by their abundance in zebrafish at the time-point they were significantly different from the control is a scale from -4 fold (dark green) to +4 fold (dark red). Where significant differences were found at more than one time-point, the colour overlay shows expression at the first instance. Dark blue squares denote 'binding', and light blue squares 'expression'; green squares stand for 'regulation', green diamonds for 'metabolism', and green circles for 'promoter binding'. Arrow heads indicate directionality of the interaction where annotated. All nodes and edges can be further interrogated by selecting the relative area of the image. [file 1471-2164-11-553-S2.zip › PathwayArchitect Zn xs DIN/1755826.html]

# REGULATION:

|  |  |
| --- | --- |
| Type | REGULATION |
| Effect | None |


---

|  |  |
| --- | --- |
| Score | 0 |


---

|  |  |
| --- | --- |
| Reference Count | 6 |


---

|  |  |
| --- | --- |
| Mechanism | Unknown |


---

|  |  |
| --- | --- |
| Reference:0 || Sentence | Shk1 bioluminescence was most sensitive to Cd and Zn, followed by Cu, and then Ni in order of decreasing sensitivity. |
| Year | 2004 |
| PMID | 14675654 |
| Journal | Water Res |
| RefScore | 1 |
| Source | PArchNLP |
  |
|  |  |


---

|  |  |
| --- | --- |
 Reference:1 || Sentence | OBJECTIVE: To observe the effects of high level Zn intake on zinc (Zn) copper (Cu), lipids metabolism and antioxidation function in man so as to provide scientific basis for Zn supplementation. |
| PMID | 15727190 |
| Year | 2004 |
| Species | Human |
| Journal | Wei Sheng Yan Jiu |
| RefScore | 1 |
| Source | PArchNLP |
  ||  |  |


---

|  |  |
| --- | --- |
 Reference:2 || Sentence | Adsorption capacity at pH 2 followed the order Cr>Cu>Pb>Cd and at pH 7 Cd>Zn, with precipitation affecting Cu and Pb behaviour. |
| PMID | 15788192 |
| Year | 2005 |
| Journal | Environ Int |
| RefScore | 0 |
| Source | PArchNLP |
  ||  |  |


---

|  |  |
| --- | --- |
 Reference:3 || Sentence | AIM: The aim of the present study was to analyze the effect of zinc on the activity of Cu/ZnSOD, lipid profile and arterial blood pressure in male Wistar rats. |
| PMID | 16152771 |
| Year | 2005 |
| Species | Rat |
| Journal | Folia Med (Plovdiv) |
| RefScore | 1 |
| Source | PArchNLP |
  ||  |  |


---

|  |  |
| --- | --- |
 Reference:4 || Sentence | The chemical partitioning patterns of Pb, Zn and Cu indicated that Pb was largely associated with the residual and NH(2)OH HCl extractable fractions. |
| PMID | 16528598 |
| Year | 2006 |
| Journal | Environ Geochem Health |
| RefScore | 2 |
| Source | PArchNLP |
  ||  |  |


---

|  |  |
| --- | --- |
 Reference:5 || Sentence | Soils around the mine were more polluted with Zn and Pb, followed by Cd and Cu. |
| PMID | 16528591 |
| Year | 2006 |
| Species | Human |
| Journal | Environ Geochem Health |
| RefScore | 1 |
| Source | PArchNLP |
  |


---

|  |  |
| --- | --- |
